# Worldwide research trends on bone metastases of lung cancer: a bibliometric analysis

**DOI:** 10.3389/fonc.2024.1429194

**Published:** 2024-10-25

**Authors:** Zhongying Rui, Dongyan Lu, Lijuan Wei, Jie Shen

**Affiliations:** Department of Nuclear Medicine, Tianjin First Central Hospital, Tianjin, China

**Keywords:** lung cancer, bone metastasis, bibliometrics, hotspot, pathogenic mechanism

## Abstract

**Background:**

Lung cancer has the highest fatality rate among all malignancies worldwide. Within this disease, bone metastasis (BM) emerges as a particularly deleterious site of metastatic dissemination, marked by a dismal prognosis. The objective of this investigation is to shed light on the current international research efforts and the development trajectory on lung cancer BM through a bibliometric analysis (performance and visualization analysis).

**Method:**

Data were obtained from the Web of Science Core Collection repository on lung cancer BM from 1 January 2012 to 1 January 2022. Subsequently, the collected data underwent scrutiny using the VOSviewer software to reveal patterns of co-authorship, co-citation, and keyword analysis, while the CiteSpace software facilitated the generation of keyword cluster maps and performed burst analyses.

**Results:**

The study included 327 papers of 2,154 authors, 587 organizations, and 41 countries, and explored the cooperation between them and the relationships between citations. Over the past decade, published papers showed a steady growth trend. China had the highest production with 189 papers, and USA had the highest collaboration with other countries, with 43 total link strength. *Lung Cancer* exhibited the highest frequency of co-cited journals, with a co-citation time of 412 and an IF/JCR partition of 6.081/Q1 in 2021. The most frequently co-cited article, authored by Tsuya A and published in *Lung Cancer* in 2007, amassed 70 co-citations. High-frequency keywords were categorized into four clusters: pathogenesis, treatment and clinical manifestations, prognosis, and diagnosis. In recent years, there has been a significant increase in the strong citation burst strength of keywords such as “predictor,” “skeletal-related events,” “efficacy,” “migration,” “docetaxel,” and “impact.” Lung adenocarcinoma is the most common type of tumor.

**Conclusion:**

This bibliometric study provides a comprehensive analysis of lung cancer BM in the recent 10 years. The field of early diagnosis, pathogenesis, and new treatments is entering a phase of rapid development and remains valuable for future research.

## 1 Introduction

Lung cancer stands as the malignancy with the highest fatality rate globally, claiming an estimated 1.8 million lives (18%). It represents the most prevalent neoplasm and ranks as the leading cause of cancer-related mortality in men, as well as the second most frequently diagnosed cancer in women (1). Non-small cell lung cancer (NSCLC) constitutes 80% of the histological variants of lung cancer (2). Advanced NSCLC has the ability to metastasize to different anatomical sites, with bones being the main site for distant metastatic spread. Bone metastases (BMs) represent one of the most harmful manifestations of metastatic lung cancer, carrying an unfavorable prognosis (3, 4). Approximately 30% to 40% of lung cancer patients experience the onset of BM during the disease progression (5). A recent large retrospective epidemiological study in China found that 17.42% of lung cancer patients had synchronous BM, the median survival time was 11.53 months, and the 1-year, 2-year, and 5-year overall survival (OS) rates were 51%, 17%, and 8%, respectively (6).

Based on the high morbidity and mortality of BM in lung cancer, researchers around the world have made many efforts and explorations. Bibliometrics is an emerging discipline that conducts quantitative research on relevant literature of specific topics in recent years (7–9). It makes statistics on research results, uses mathematical methods to conduct quantitative analysis on literature, analyzes key research fields, understands research quality, and predicts future research directions (10, 11). This study utilizes the Science Citation Index Expanded (SCI-E) database to evaluate literature on BM in lung cancer from 2012 to 2022. This study aims to examine the conceptual framework supporting innovative research, identify key areas of interest, and outline the evolutionary paths of BM in lung cancer over the last decade through bibliometric analysis.

## 2 Research methods and data sources

Our study was a retrospective bibliometric analysis that did not involve human subjects and was exempt from an institutional review board or ethics committee approval.

### 2.1 Research methods

Bibliometrics is an independent discipline and has become widely applied in literature analysis (12). The bibliometric analysis provides a quantitative method, including performance analysis (13) and visualized analysis (14), for reviewing and investigating extant literature in a given field (15). Thus, it could help excavate the internal relationship of details in literature.

Export the comprehensive records of the search outcomes and associated references in TXT format as a file for further analysis. Bibliometric analysis of the co-authorship including country, organization, and author; the co-citation including reference, journal, and author; as well as keyword analysis were used by VOSviewer software. Use the CiteSpace software (version 6.1.R3 basic) to construct a keyword cluster map, and use the CiteSpace software (version 6.2.R1 basic) to conduct burst analysis.

### 2.2 Data sources

**Figure 1:**
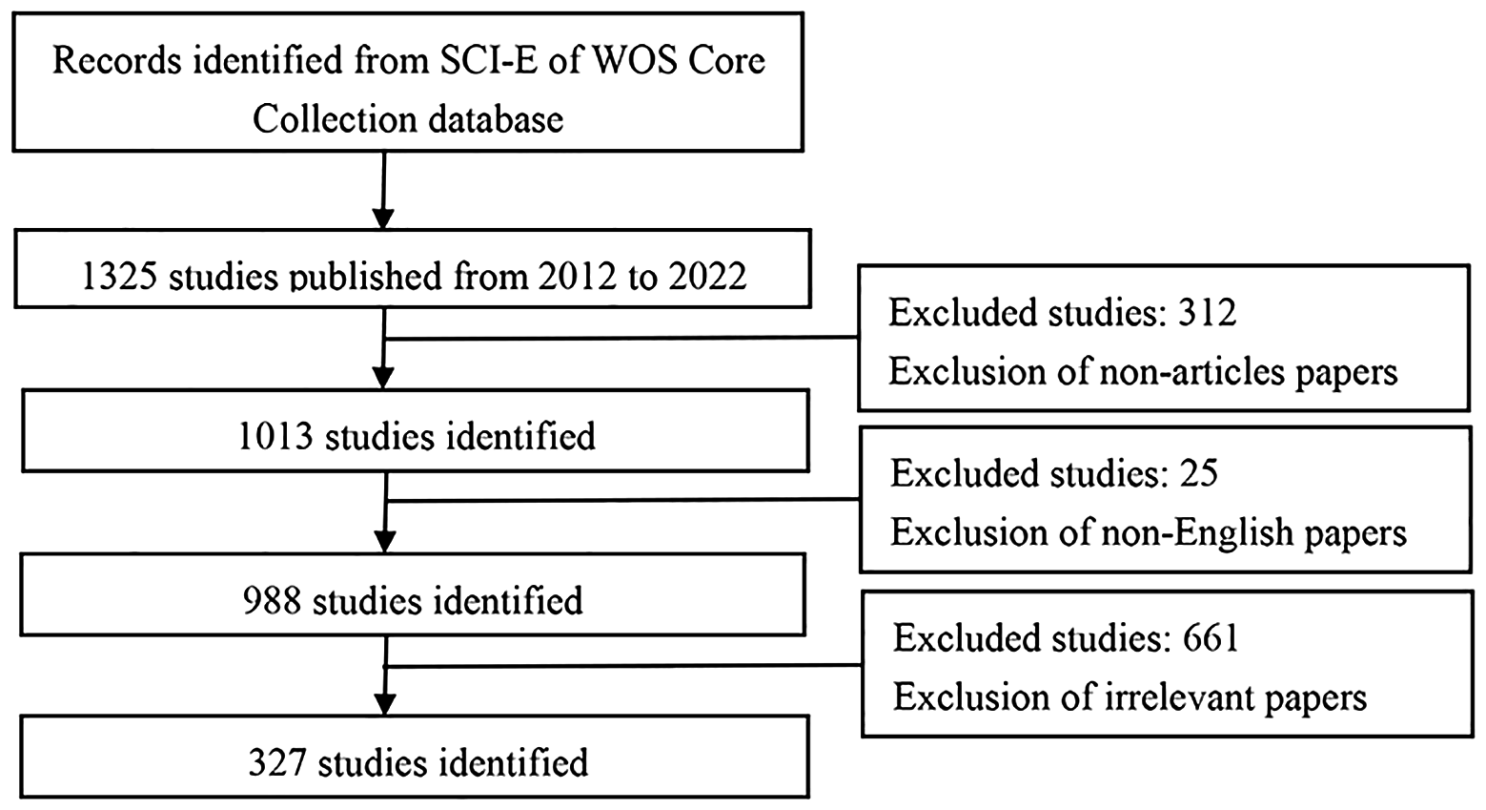
Flow chart of literature screening related to lung cancer bone metastasis research.

We used the SCI-E of Web of Science Core Collection (WoSCC) bibliographic database to perform bibliometric analysis. The publication period in this study was set from 1 January 2012 to 1 January 2022. The search terms were presented as follows: TS= ((“lung cancer” OR “lung carcinoma”) AND (“bone metastas*” OR “skeletal metastas*”)) OR TS = “lung cancer bone metastas*” OR TS = “bone metastas* from lung cancer”. In light of the swift database updates, literature retrieval was executed within a single day (14 October 2022) to mitigate potential discrepancies. The document was classified as an “article,” with English selected as the language. A total of 327 articles were analyzed for this study. The meticulous screening process is outlined in **Figure 1**.

### 2.3 Statistical processing

The study describes the current situation of research on lung cancer BM using absolute numbers, trend, and the distribution of changes. It does not involve comparisons between groups.

## 3 Results

### 3.1 Literature search results

The 327 papers used in this study were written by 2,154 scholars from 587 institutions across 41 countries. These papers were disseminated across 174 scholarly journals and referenced 7,103 sources obtained from 1,609 distinct journals.

**Figure 2:**
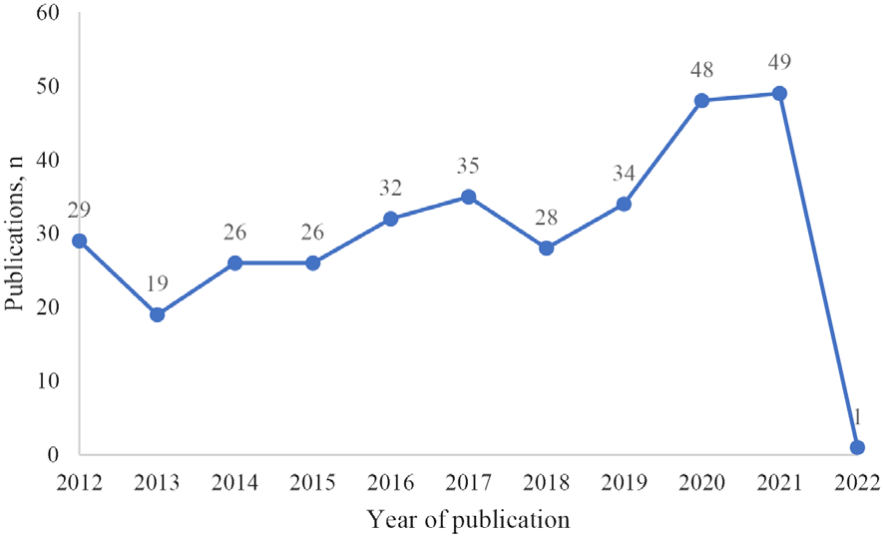
Scientific publications per year.

The annual number of published papers is an important value to evaluate the development of scientific research. Before 2018, the number of published documents increased slowly, but after 2018, the number of published documents increased rapidly. In general, the research on BM of lung cancer showed a steady growth trend, reflecting the increasing popularity of this research (**Figure 2**).

### 3.2 Bibliometric analysis of the co-authorship

**Figure 3:**
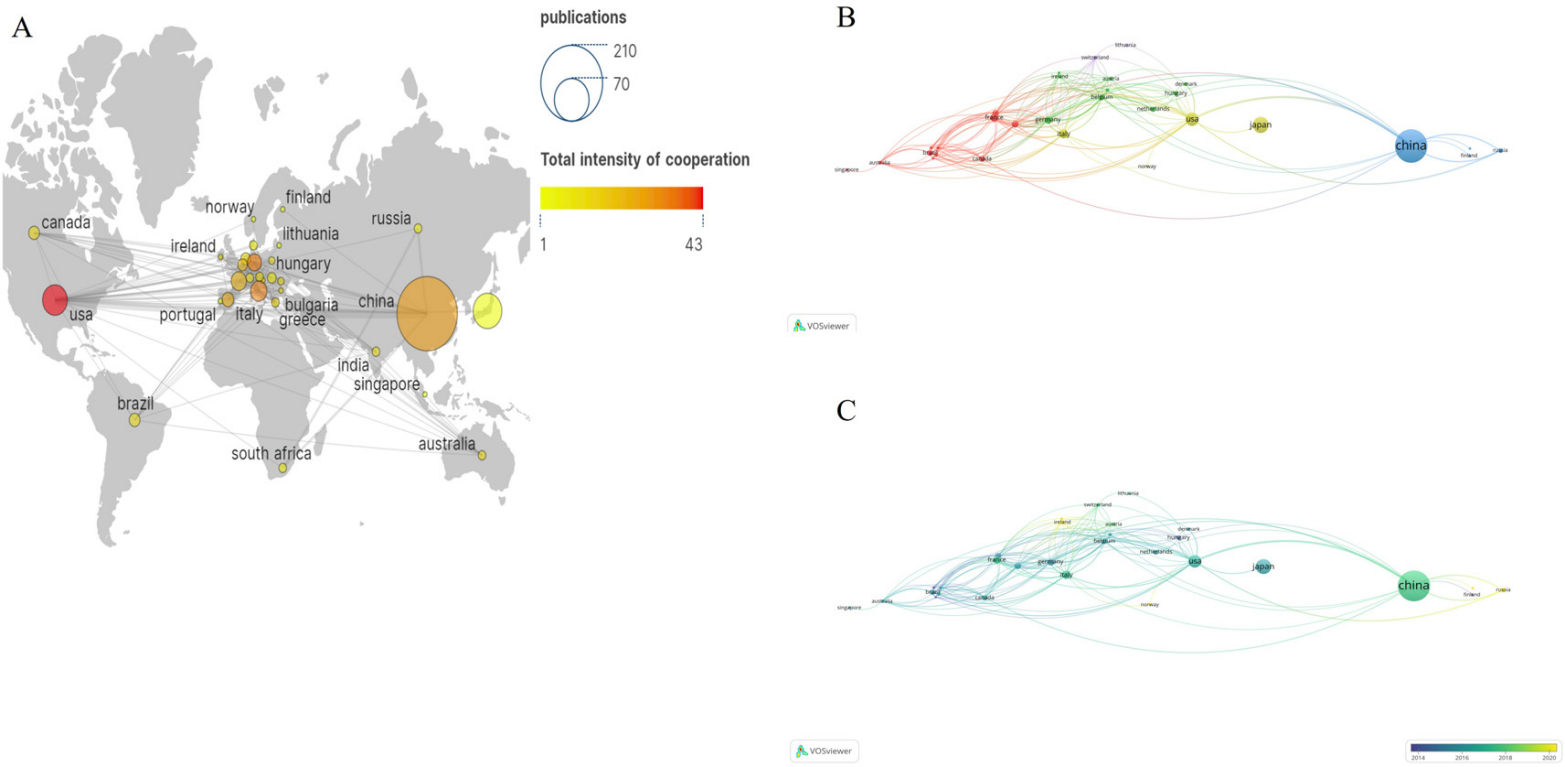
The bibliometric analysis of the co-authorship of countries/regions in the field of lung cancer bone metastasis. **(A)** Country-scientific publications and total intensity of cooperation in the field of lung cancer bone metastasis. **(B)** The network visualization map of countries/regions collaborations in the field of lung cancer bone metastasis. **(C)** The overlay visualization map of countries/regions collaborations in the field of lung cancer bone metastasis.

Co-authorship could strengthen communication and actively identify and solve potential problems. **Table 1** outlines the top five countries in publication count: China, Japan, USA, Italy, and South Korea. China exhibited the most prolific output, generating 189 papers and engaging in collaborative efforts with other nations, resulting in a total of 29 total link strength related to lung cancer BM. USA had the highest collaboration with other countries, which published 32 papers and 43 total link strength on lung cancer BM. As depicted in **Figures 3A, B**, the size of the data points corresponds to the number of publications, while the color spectrum signifies the overall intensity of collaboration. **Figure 3C** illustrates a temporal analysis network among these countries, with the varying hues indicating their average years of activity, ranging from deep blue to light blue and from pale yellow to deep yellow. Hungary, Finland, Poland, and Bulgaria were earlier studied lung cancer BM. Since 2016, an increasing number of countries have shown a strong interest in lung cancer BM. There is an upward trajectory in the collaboration among nations.

**Table 1:** The top five most active countries related to lung cancer bone metastasis.

Rank	Country	Documents	Citations	Average Citation/Publication
**1**	China	189	2,215	11.72
**2**	Japan	43	413	9.60
**3**	USA	32	887	27.72
**4**	Italy	14	552	39.43
**5**	South Korea	14	167	11.93

**Figure 4:**
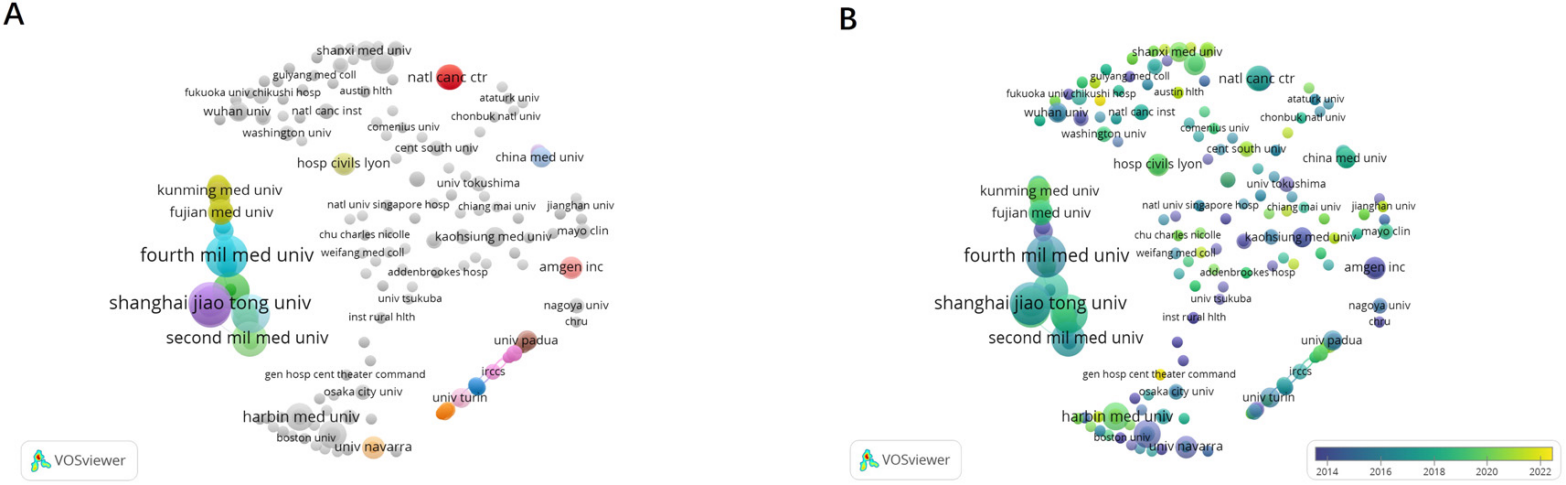
The bibliometric analysis of the co-authorship of organizations in the field of lung cancer bone metastasis. **(A)** The network visualization map of collaborations among organizations in the field of lung cancer bone metastasis. **(B)** The overlay visualization map of collaborations among organizations in the field of lung cancer bone metastasis.

The list (**Table 2**) shows the top 10 organizations with the number of publications. Shanghai Jiaotong University led in research output by publishing 14 relevant papers, receiving 210 citations, and establishing a total link strength of 14. As shown in **Figure 4**, the research in this field was relatively scattered among institutions and there was little cooperation between these institutions. **Figure 4B** illustrates the temporal overlap analysis network of these institutions. The color gradient, transitioning from deep blue to light blue and from pale yellow to deep yellow, signifies the average years of activity among these organizations.

**Table 2:** The top 10 most active organizations related to lung cancer bone metastasis.

Rank	Organization	Documents	Citations	Average Citation/Publication
**1**	Shanghai Jiaotong Univ	14	210	15
**2**	Fourth Mil Med Univ	13	188	14.46
**3**	Fudan Univ	12	81	6.75
**4**	Sichuan Univ	11	126	11.45
**5**	Sun Yat Sen Univ	10	110	11
**6**	Second Mil Med Univ	9	173	19.22
**7**	Tongji Univ	7	52	7.43
**8**	Shandong Univ	6	114	19
**9**	Hebei Med Univ	6	93	15.5
**10**	Harbin Med Univ	6	33	5.5

**Figure 5:**
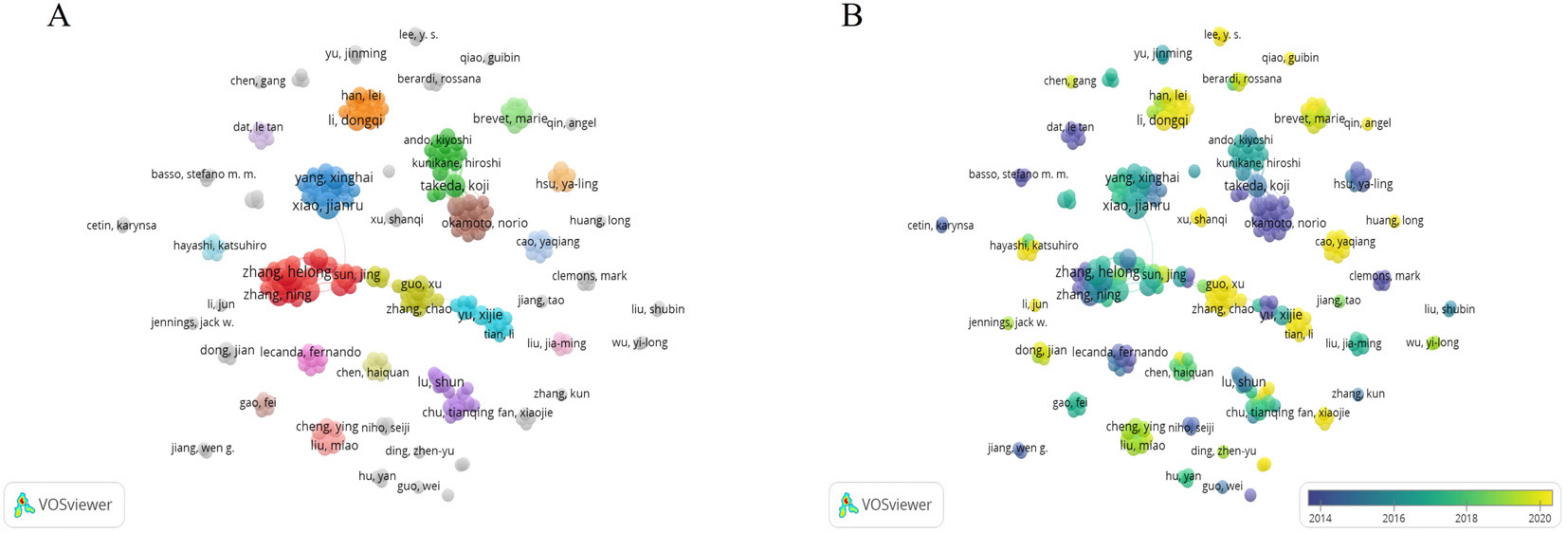
The bibliometric analysis of the co-authorship of authors in the field of lung cancer bone metastasis. **(A)** The network visualization map of collaborations among authors in the field of lung cancer bone metastasis. **(B)** The overlay visualization map of collaborations among authors in the field of lung cancer bone metastasis.

The list (**Table 3**) shows the top 10 authors with the number of publications, mainly from China. There were few links between authors, and the related literature published by each author was also few (**Figure 5**).

**Table 3:** The top 10 most active authors related to lung cancer bone metastasis.

Rank	Author	Documents	Citations	Average Citation/Publication
**1**	Xiao, J.R.	7	162	23.14
**2**	Zhang, H.L.	7	85	12.14
**3**	Pang, H.L.	6	81	13.5
**4**	Liu, L.L.	6	73	12.17
**5**	Yang, X.H.	5	117	23.4
**6**	Zhou, W	5	117	23.4
**7**	Zhang, N	5	97	19.4
**8**	Takeda, K	5	73	14.6
**9**	Ma, N.Q.	5	67	13.4
**10**	Shen, W.W.	5	53	10.6

### 3.3 Bibliometric analysis of the co-citation

**Figure 6:**
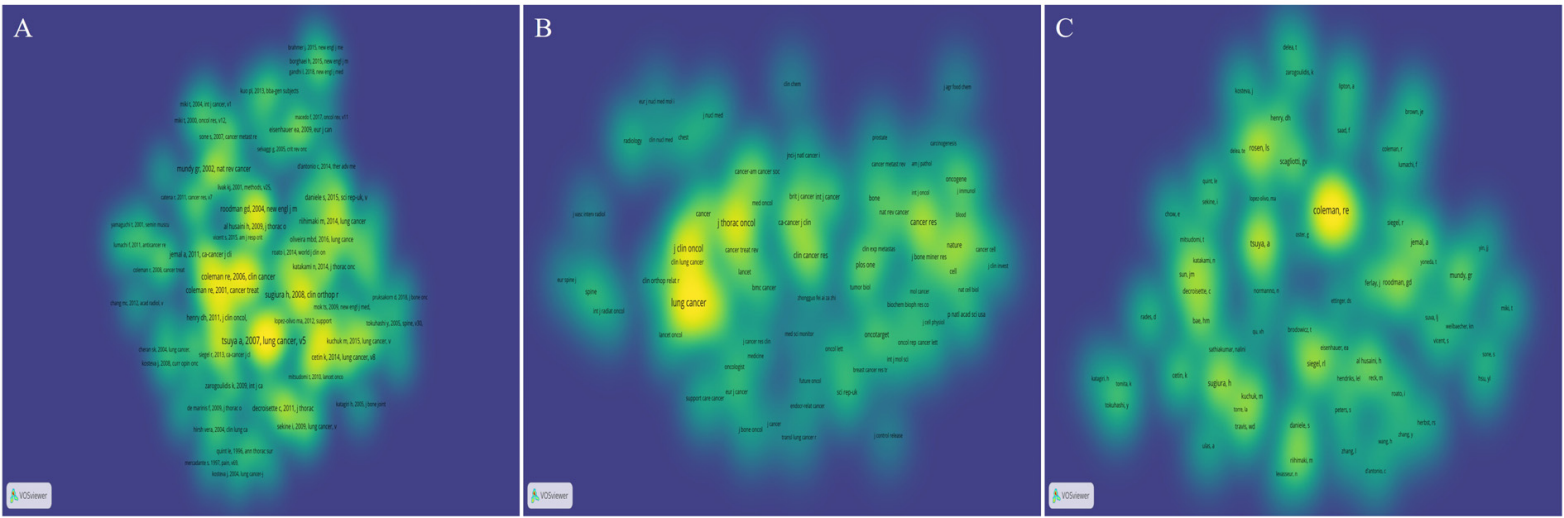
The bibliometric analysis of the co-citation in the field of lung cancer bone metastasis. **(A)** The density visualization map of references. **(B)** The density visualization map of journals. **(C)** The density visualization map of authors.

The publications most frequently cited within a specific field are indicative of the research’s impact. Utilizing VOSviewer, co-citation analysis was conducted to identify references cited more than 5 times, journals cited more than 20 times, and authors cited more than 10 times. This process resulted in the selection of 125 papers, 86 journals, and 72 authors for density visualization mapping, as illustrated in **Figures 6A–C**, respectively.

The list (**Table 4**) shows the top five co-cited references. These articles were published between 2002 and 2008 and have received over 35 citations. Among them, the most frequently cited article was titled “Skeletal metastases in non-small cell lung cancer: a retrospective study (16)”, which was published by Tsuya A in *Lung Cancer* in 2007, with 70 co-citations. The second most cited publication was entitled “Clinical features of metastatic bone disease and risk of skeletal morbidity (17)”, published by Coleman RE in *Clin Cancer Res* in 2006, with 47 co-citations. The list (**Table 5**) shows the top five co-cited journals. Among these, *Lung Cancer* emerged as the primary journal of co-citations, with a co-citation count of 412 and an IF/JCR partition of 6.081/Q1 in 2021. Other notable journals included *J Thorac Oncol*, *J Clin Oncol*, *New Engl J Med*, and *Cancer Res*. The list (**Table 6**) shows the top five co-cited authors, namely, Coleman RE (144), Rosen LS (64), Tsuya A (70), Sugiura H (50), and Roodman GD (40).

**Table 4:** The top five co-cited references related to lung cancer bone metastasis.

Rank	Article title	Citations	Total link strength
**1**	Skeletal metastases in non-small cell lung cancer: a retrospective study	70	62
**2**	Clinical features of metastatic bone disease and risk of skeletal morbidity	47	41
**3**	Predictors of survival in patients with bone metastasis of lung cancer	50	40
**4**	Long-term efficacy and safety of zoledronic acid in the treatment of skeletal metastases in patients with non-small cell lung carcinoma and other solid tumors: a randomized, phase III, double-blind, placebo-controlled trial	36	36
**5**	Metastasis to bone: causes, consequences and therapeutic opportunities	35	19

**Table 5:** The top five co-cited journals related to lung cancer bone metastasis.

Rank	Journal	Citations	Total link strength
**1**	Lung Cancer	412	1,645
**2**	J Thorac Oncol	305	1,538
**3**	J Clin Oncol	283	1,374
**4**	New Engl J Med	193	950
**5**	Cancer Res	203	443

**Table 6:** The top five co-cited authors related to lung cancer bone metastasis.

Rank	Author	Citations	Total link strength
**1**	Coleman, RE	144	193
**2**	Rosen, LS	64	143
**3**	Tsuya, A	70	126
**4**	Sugiura, H	50	71
**5**	Roodman, GD	40	59

### 3.4 Keyword analysis

**Figure 7:**
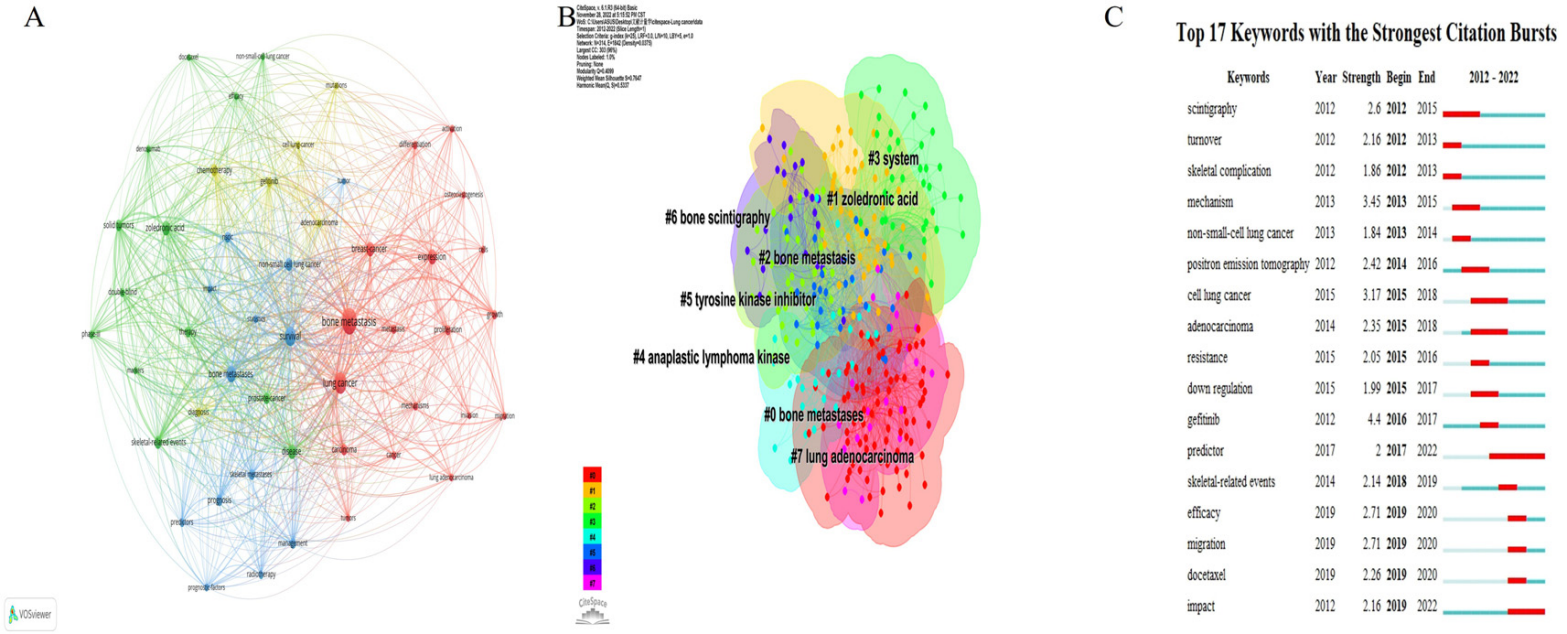
The analysis of keywords in the field of lung cancer bone metastasis. **(A)** The network visualization map of keywords. **(B)** The keyword cluster map. **(C)** Burst analysis showed that the utility of key words changed annually.

A total of 1,375 keywords occurred on lung cancer BM. A network visualization map consisting of 50 prominent keywords, each appearing more than 10 times, was generated (**Figure 7A**). The size of the nodes represents the frequency of keyword usage, while their color denotes the respective keyword cluster. Furthermore, the proximity of nodes indicates the strength of their correlation, with closely related keywords clustered together. The 50 keywords were categorized into four distinct clusters: Cluster 1, highlighted in red, encompassed 18 keywords focusing primarily on the pathogenesis of lung cancer BM, such as “expression”, “proliferation”, and “osteoclastogenesis”. Cluster 2, depicted in green, comprised 13 keywords emphasizing various treatment modalities for lung cancer BM, such as “zoledronic acid” and “denosumab,” along with clinical symptoms such as “skeletal-related events.” Cluster 3, represented in blue, comprised 13 keywords centered around prognostic indicators such as “prognostic factors,” “predictors,” and “survival.” Finally, Cluster 4, highlighted in yellow, contained six intriguing keywords that focused on tumor pathological types, such as “adenocarcinoma” and “cell lung-cancer”.

CiteSpace software was used to construct a keyword cluster map and burst analysis. As shown in **Figure 7B**, we proceeded to conduct a clustering analysis on these co-occurring keywords, resulting in their categorization into eight distinct clusters: Cluster 1 (bone metastases), Cluster 2 (zoledronic acid), Cluster 3 (bone metastasis), Cluster 4 (system), Cluster 5 (anaplastic lymphoma kinase), Cluster 6 (tyrosine kinase inhibitor), Cluster 7 (bone scintigraphy), and Cluster 8 (lung adenocarcinoma). These clusters epitomize the paramount themes in lung cancer BM research, highlighting the predominant focus on pathogenesis, treatment and clinical manifestations, prognosis, and diagnosis. **Figure 7C** displays the top 17 keywords with the strongest citation bursts. The keywords such as “predictor” (burst duration from 2017 to 2022), “skeletal-related events” (2018–2019), “efficacy” (2019–2020), “migration” (2019–2020), “docetaxel” (2019–2020), and “impact” (2019–2022) have recently garnered significant interest, underscoring their status as prevalent research topics in recent years and likely into the foreseeable future.

## 4 Discussion

Lung cancer with BM has high morbidity and mortality. It is necessary to analyze the related research to understand the overall situation of the current related research. Web of Science (WOS), esteemed as a premier digital repository of scholarly literature, stands as a cornerstone resource for researchers across diverse domains for bibliometric analysis. Hence, it was judiciously chosen as the primary data reservoir for this investigation (18–20). Employing the method of bibliometric analysis, this study scrutinized the current research landscape and potential trajectories related to lung cancer BM. Keyword analysis revealed key areas and emerging trends in the field of lung cancer BM, highlighting the main research themes including pathogenesis, treatment options, prognostic factors, and diagnostic methods.

### 4.1 Pathogenic mechanism

#### 4.1.1 Cytokine

Tumor cells stimulate osteoclasts to release the ligand interleukin (IL)-19 for the IL receptor 20 subunit β (IL-20RB), which then triggers IL-20RB-expressing tumor cells to initiate downstream JAK1/STAT3 signaling. This activation culminates in increased proliferation of tumor cells within the bone. The IL-19/IL-20RB/STAT3 axis plays a pivotal role in orchestrating the direct pro-tumoral function of the osteoclastic niche (21). The identified IL-19/IL-20RB/STAT3 axis presents various opportunities for targeting treatment. For instance, targeting intracellular STAT3 with napabucasin, a drug clinically approved for treating gastric cancer and under clinical trials for more aggressive malignancies (22, 23), has also shown promise in alleviating lung cancer BM. Furthermore, inhibiting IL-20RB with a neutralizing antibody significantly attenuated lung cancer BM (21). MicroRNA-182 (miR-182) promotes NSCLC cells to secrete IL-8, thereby enhancing osteoclastogenesis through the activation of STAT3 signaling (miR-182/IL-8/STAT3 axis) in osteoclast progenitor cells (24).

The proteolytically generated component of the complement cascade, C5a, engages its cognate receptor (C5aR1), eliciting diverse cellular reactions implicated in oncogenic progression. C5aR1 is expressed across a spectrum of immune and neoplastic cell lineages, including lung carcinoma cells. The mechanistic implication of the C5a/C5aR1 axis in the skeletal tropism of lung cancer underscores its potential as an innovative therapeutic approach for managing BM in lung cancer (25).

Heightened expression of epidermal growth factor-like domain multiple 6 (EGFL6), a secretory protein, in lung adenocarcinoma cells amplifies their proliferative, migratory, and invasive capacities. This elevation fosters epithelial–mesenchymal transformation (EMT) and triggers the Wnt/β-catenin and PI3K/AKT/mTOR cascades. Furthermore, EGFL6 secretion from human lung adenocarcinoma cells enhances the differentiation of osteoclasts from bone marrow mononuclear macrophages (BMMs) in murine models. This process is mediated by the NF-κB and c-Fos/NFATc1 pathways (26). Activation triggered by bone sialoprotein (BSP) on matrix metalloproteinase (MMP)-14 enhances the migratory and invasive potential of lung carcinoma cells through the PI3K/AKT/AP-1 axis (27).

#### 4.1.2 Chemokines

Chemokines, small signaling proteins ranging from 8 to 10 kDa, are secreted by cells and serve as pivotal mediators not only in inflammation but also in tumor initiation, growth, infiltration, and dissemination (28). Lung cancer cells express specific chemokines (such as CXCL12/14/16, CCL7/22, CX3CL1, and XCL1) and their corresponding receptors. This expression can drive tumor cell infiltration into bone tissue, disrupting the delicate equilibrium of the bone microenvironment and inflicting severe detrimental effects on bone integrity. Certain chemokines, specifically designed for the bone marrow environment in lung cancer, coordinate the proliferation, viability, and activation of harmful feedback loops within both lung cancer cells and the bone tumor microenvironment (TME), thus initiating and sustaining BM (29). For instance, in lung cancer cell metastasis, CXCL16 can exist as membrane-bound CXCL16 and soluble CXCL16. The former induces tumor immunity by promoting cell adhesion and lymphocyte aggregation at tumor sites, while the latter participates in processes related to cancer cell proliferation and metastasis by regulating mitosis and anti-apoptosis. The migratory and invasive capabilities of lung adenocarcinomas and squamous cell carcinomas are governed by ADAM10. Discrepancies between the two lung cancer subtypes are attributed to the variant expression of MMPs following CXCL16 stimulation (30). Although the interaction between CCL22 and its receptor CCR4 may recruit regulatory T cells (Tregs) into the TME, thereby bolstering tumor immune evasion, it also fosters the infiltration of anticancer tumor-infiltrating lymphocytes (TILs), presenting a dual effect (29). Consequently, chemokines and corresponding receptors can enhance immune responses and affect clinical outcomes by determining the distribution and matrix composition of immune cells in the TME. Regulatory strategies targeting chemokines and corresponding receptors should continue to be expanded in future research.

#### 4.1.3 Seed and soil hypothesis

The bone marrow microenvironment comprises a plethora of cellular constituents, including bone marrow adipocytes (BMAs), osteoblasts (OBs), osteoclasts (OCs), endothelial cells (ECs), and immune cells. Notably, BMA constitutes approximately 70% of the adult bone marrow volume. Cumulative investigations underscore the pivotal role of BMA as a crucial energy reservoir for tumor cells, facilitating their proliferation and migratory capabilities. Moreover, BMA exerts regulatory influence over osteogenesis, OC activity, and immune responses within the TME. This influence is mediated through the secretion of adipokines, cytokines, and inflammatory mediators. Adipokines such as leptin and adiponectin have been shown to enhance lung cancer metastasis (31). NSCLCs associated with chronic inflammation and external stimuli, such as cigarette smoke and environmental toxins, can alter the phenotypes of tumor-associated macrophages (TAMs), impairing their effector functions. Mounting evidence suggests that tissue-resident AMs (TRAMs) exhibit distinctive transcriptional and epigenetic profiles compared to monocyte-derived AMs (MoAMs), which may contribute to TAM heterogeneity and exert varied functions. These disparities underscore the complex interplay between myeloid cells and their surroundings. Identifying pivotal regulatory factors governing these variances will enhance our comprehension of macrophage diversity and pave the way for novel therapeutic strategies in the management of lung cancer (32).

Crucially, the interaction between tumor cell surface receptors and bone marrow and bone stromal cells significantly impacts bone marrow development. These interactions promote tumor growth by releasing abundant amounts of growth factors, cytokines, and angiogenic factors. Concurrently, they increase OC activity and enhance bone resorption. As explained by the seed and soil hypothesis, the distinctive characteristics of tumors and the supportive bone microenvironment determine the preference for bone as a site for metastasis (33). The pathogenic mechanism in BM of lung cancer is caused by multiple factors, which needs further study and analysis. The understanding of the pathogenic mechanism determines the research of new treatments.

### 4.2 Therapeutic method

Lung cancer spreads metastases through three distinct phases: tumor infiltration, tumor cell dissemination, and infiltration and metastasis to bone tissue (34). Predominant sites of metastasis include the spinal column, ribs, femur, and sternum. Based on the lesion’s distinctive imaging characteristics and the prevalence of osteoblasts/osteoclasts, metastases are categorized into osteolytic, osteogenic, or hybrid phenotypes (35). Skeletal-related events (SREs) induced by BM is a direct factor affecting the quality of life and survival of patients. In addition to symptomatic treatment for pain relief, there are the following treatments.

#### 4.2.1 Chemotherapy

In recent years, innovative therapeutic approaches, including targeted therapies, immunotherapies, and antibody–drug conjugates (ADCs), have emerged for the treatment of lung cancer. Nevertheless, chemotherapy remains a critical component, serving both as a key partner to these new therapies and as a fallback option in cases of drug resistance. A study conducted by Han et al. systematically analyzed randomized controlled trials (RCTs) evaluating the combination of bevacizumab with platinum-based chemotherapy for advanced NSCLC across six major databases. The findings revealed that the combination therapy significantly improved antitumor efficacy compared to chemotherapy alone, as evidenced by higher objective response rates (ORRs), disease control rates (DCRs), and 1-year, 2-year, and 3-year survival rates (36).

Platinum-containing dual drug combination chemotherapy is the standard first-line regimen for patients with advanced driver-negative lung cancer and is significantly superior to single-drug regimen in terms of response rate and survival. Cisplatin- or carboplatin-based dual drug regimen containing platinum is recommended (37).

Platinum-based chemotherapy remains the cornerstone of lung cancer treatment. Depending on the subtype of lung cancer, it is often combined with a second chemotherapeutic agent to create a two-drug regimen. For example, paclitaxel is commonly used in conjunction with platinum compounds for NSCLC (38), while etoposide is paired with platinum for SCLC (39).

#### 4.2.2 Radiation therapy

Targeted radiopharmaceutical therapy aimed at bone offers a unique advantage in relieving the diffuse pain associated with osteoblastic BM. For example, ^89^Sr is a bone-friendly radionuclide with biochemical properties similar to calcium. After intravenous injection, the blood can quickly clear and selectively concentrate in the active bone formation site, especially the BM, which can reduce the radiation effect on normal bone tissue, shrink or kill the BM, reduce the activity of alkaline phosphatase, reduce osteolysis, and promote bone repair. Reduce the pressure on the endosteum and the perineum, thereby relieving local pain. The half-life of ^89^Sr in BM can reach 50 days, and it can exert its drug effect sustainably, maintain the therapeutic effect, play a good role in analgesia, and reduce blood calcium level (40). Radium-223 (^233^Ra) is a novel bone-targeting radioisotope injected intravenously that emits alpha particles. In the current study of BM in prostate tumors, both OS and the time to first onset of symptomatic SREs have been improved (41, 42). However, the efficacy of lung cancer in patients with BM remains to be observed.

It also includes external radiation therapy, which is the first choice for palliative radiotherapy of lung cancer BM (43).

#### 4.2.3 Molecular targeting treatment

In a keyword cluster map, our study found the Cluster “tyrosine kinase inhibitor (TKI)”. TKI can reduce the differentiation of osteoclasts by regulating bone marrow stromal cells (BMSCs), thereby delaying the bone destruction of tumor BM, thereby reducing SREs and prolonging the time of the first SREs (44). Gefitinib, an epidermal growth factor receptor TKI (EGFR-TKI) that targets mutations of the EGFR, can inhibit bone reabsorption and significantly improve the occurrence of pathological fractures while exerting antitumor therapy (45). Also included are crizotinib, an inhibitor of anaplastic lymphoma kinase (ALK), MET and ROS-1 tyrosine kinases, and bevacizumab, a humanized monoclonal antibody against vascular endothelial growth factor receptor (VEGFR) (37).

#### 4.2.4 Immunotherapy

The anti-PD-1 antibodies nivolumab and pembrolizumab interact with the PD-1 receptor on T cells, while anti-PD-L1 antibodies can engage with either the PD-L1 receptor on immune cells or tumor cells. This interaction effectively impedes the inhibitory effect of the PD-1/PD-L1 pathway on T cells, thereby promoting an antitumor response (46, 47).

It also includes bisphosphonate bone repair, traditional Chinese medicine (TCM), and surgery. Bisphosphonates can prevent bone pain, pathological fractures, spinal cord compression, hypercalcemia, and other SREs in patients with lung cancer with or without symptoms of BM. They are considered the primary medication for managing BM in lung cancer and can be used in conjunction with conventional antitumor therapies to control BM by inhibiting bone resorption (48). TCM is characterized by its novel pharmacological mechanisms, low toxicity, and minimal side effects. It encompasses numerous active compounds, including flavonoids, alkaloids, terpenoids, and polyphenols, which have demonstrated efficacy against lung cancer (49). Surgical treatment for BM is usually used only for lesions with complete or pathological fracture tendencies, and the surgical intervention should be consistent with treatment objectives (50).

In conclusion, adopting a multiple department treatment (MDT) model and formulating an individualized comprehensive treatment plan in a planned and reasonable way will help to improve the quality of life of patients.

### 4.3 Diagnosis and prognosis

It is imperative to promptly detect BM, not only for staging and prognostication but also for initiating preventive and therapeutic interventions that could potentially reduce morbidity and mortality. Clinically, the diagnosis of BM originating from lung cancer primarily relies on clinical manifestations and various imaging modalities such as plain radiographs, CT scans, MRI scans, SPECT/CT scans, and PET/CT scans. Once tumor metastasis progresses to the stage of radiologically evident lesions, controlling bone destruction becomes a challenging task (44).

Research by Teng et al. (51) underscored that the development of a non-invasive approach for early diagnosis could significantly enhance the prognosis and quality of life for lung cancer patients. They proposed a serum molecular diagnostic model comprising bone microenvironment cytokines (osteoprotegerin and parathyroid hormone-related peptide) as well as bone turnover markers including tP1NP, a bone formation marker, and β-CTx, a bone resorption marker. This could aid in diagnosing and monitoring the progression of BM in lung cancer. Additionally, Zhu et al.’s study (52) observed significant differences in bone metabolism markers between lung cancer patients with and without BM. However, the effectiveness of a single marker in predicting both positive and negative outcomes simultaneously is limited. They proposed a multiple regression model [p4 = 5.011 + 2.6 × 10−5(β-CTX × TPINP) + 4.10−2 ×(ALP) − 2.779(CA)] to assess lung cancer progression and guide early interventions for BM.

The research conducted by Niu et al. (53) highlighted that lung cancer patients with a T_4_N_3_ stage and positive BSP expression exhibit an increased susceptibility to BM. Furthermore, persistent smoking habits and the presence of multiple BM are significantly linked to a higher occurrence of SREs in lung cancer patients with BM. In patients with lung adenocarcinoma undergoing surgical treatment, the levels of EGFL6 in tumor tissues are correlated with the presence of BM and TNM staging (26). In the investigation by Liu et al. (54), an extensive literature review was conducted, encompassing multiple common databases up to 4 September 2022. Their findings revealed that the presence of BM serves as a predictor of poor OS in NSCLC patients treated with immune checkpoint inhibitors (ICIs). However, the progression-free survival (PFS) does not appear to be affected by the presence of BM. By employing single-cell sequencing on lung tissue from healthy individuals and NSCLC patients with BM, brain metastasis, and intrapulmonary metastasis, Xu et al. identified a subpopulation of antigen-presenting fibroblasts (apCAF) prevalent in NSCLC cases with BM. Subsequent analysis of intercellular signaling networks suggests that apCAF may contribute to BM by activating cancer stemness-related signaling pathways including SPP1-CD44/PTGER4 (55).

In the study, we found that although the overall trend is increasing, there are not many publications on BM in lung cancer, with only 327 papers. While China had the highest number of relevant publications, USA showed the most robust collaboration with other nations. Despite the greater volume of publications originating from Chinese authors and organizations, the interconnectedness between them remained limited. Moreover, our analysis revealed a relatively sparse body of literature authored by prominent researchers in this field. Xiao JR, who contributed the highest number of publications, only yielded seven articles. This disparity suggests a comparative lack of research activity in this field compared to others. Noteworthy journals such as *Lung Cancer*, *Journal of Thoracic Oncology*, *Journal of Clinical Oncology*, *New England Journal of Medicine*, and *Cancer Research* have received frequent citations, indicating that the literature in these journals deserves further attention. Co-cited references serve as barometers of the evolving landscape and trends in related fields, providing valuable insights for frontier analysis, domain assessment, and scientific evaluation. They provide a solid foundation for making informed decisions in scientific pursuits (7, 56). Notably, studies that are co-cited are considered to have a significant academic impact. In our investigation, the most frequently co-cited article, authored by Tsuya A and published in *Lung Cancer* in 2007, amassed 70 co-citations. Tsuya A et al.’s study (16) illuminated that the patients experiencing SREs tended to exhibit poorer survival outcomes, while no significant difference in survival was discerned between patients with and without skeletal metastases.

The current study is not without limitations. Primarily, the literature examined in this study was exclusively sourced from the WoSCC database, and only English-language articles were considered. This approach unavoidably omitted other valuable literature sources. Second, the search terms may miss some documents closely related to the field, which may have led to selection bias.

## 5 Conclusions

Lung cancer with BM has high morbidity and mortality. Over the past decade (1 January 2012 to 1 January 2022), published papers showed a steady growth trend. China had the highest production and USA had the highest collaboration with other countries. The most frequently cited article was published by Tsuya A in *Lung Cancer* in 2007, with 70 co-citations. *Lung Cancer* emerged as the primary journal of co-citations. Enhancing collaboration among authors, institutions, and global entities remains imperative. It is our fervent aspiration that an increased number of researchers and institutions will champion academic discourse and fortify partnerships to bridge the gaps in lung cancer BM. Nations should actively promote channels for communication and collaboration, providing a conducive platform for scholars and institutions, and proactively identifying and addressing potential challenges. Pathogenesis, treatment modalities, prognostic markers, and diagnostic methodologies represent burgeoning areas of inquiry and are poised to shape future research trajectories. With advancements in precise localization-based diagnostics and emerging therapeutic modalities, patients suffering from lung cancer BM are poised for an improved prognosis.

## Data Availability

The original contributions presented in the study are included in the article/supplementary material. Further inquiries can be directed to the corresponding author.
